# Personalized Digital Health Solutions to Increasing Diabetes-Related Knowledge and Behavioral Outcomes: Results From a Randomized Controlled Trial

**DOI:** 10.2196/87364

**Published:** 2026-06-01

**Authors:** Marcia Ory, Christi H Esquivel, Gang Han, Samuel D Castiglione Towne Jr, SangNam Ahn, Matthew Lee Smith

**Affiliations:** 1 Center for Community Health and Aging, School of Public Health, Texas A&M University, 212 Adriance Lab Rd, College Station, TX, 77843, United States, 1 9798201498; 2 Department of Environmental and Occupational Health, School of Public Health, Texas A&M University, College Station, TX, United States; 3 Department of Epidemiology and Biostatistics, School of Public Health, Texas A&M University, College Station, TX, United States; 4 Disability, Aging, and Technology Cluster, University of Central Florida, Orlando, FL, United States; 5 School of Global Health Management and Informatics, University of Central Florida, Orlando, FL, United States; 6 Department of Health Management and Policy, College for Public Health and Social Justice, Saint Louis University, Saint Louis, MO, United States; 7 Department of Health Behavior, School of Public Health, Texas A&M University, College Station, TX, United States; 8 Department of Applied Health Science, School of Public Health, Indiana University, Bloomington, IN, United States; 9 Center for Health by Design, School of Public Health, Indiana University, Bloomington, IN, United States

**Keywords:** diabetes self-management education and support, diabetes self-care behaviors, digital health, randomized clinical trial, diabetes knowledge, diabetes confidence, diabetes distress

## Abstract

**Background:**

The prevalence of diabetes in the United States necessitates investigations into how to better enable adults with type 2 diabetes mellitus (T2DM) to manage their health using easy-to-access and personally adaptable technologies. The ubiquity of digital content further justifies the need to consider the impact of different digital intervention modalities in diabetes self-care activities.

**Objective:**

This study aimed to compare the impact of 2 digital diabetes self-care education programs delivered separately and in combination to adults with T2DM across various settings in Texas.

**Methods:**

We conducted a randomized controlled trial in Texas with 188 adults with T2DM to assess whether 2 different interventions alone (Virtual Making Moves with Diabetes or Technology-Based Education and Support) or in combination (Virtual Making Moves with Diabetes followed by Technology-Based Education and Support) improved multiple outcomes associated with diabetes self-management. We used several estimation techniques, including generalized estimating equations, to account for multiple factors simultaneously.

**Results:**

All 3 digital intervention modalities led to statistically significant improvements in diabetes-related confidence, distress, and self-care behaviors, with significance from baseline through 6 months and supported by moderate to strong effect sizes (Cohen *d*) ranging from 0.446 to 0.827 at 3-month follow-up versus baseline and from 0.538 to 0.888 at 6-month follow-up versus baseline. No statistically significant superiority was observed among the intervention modalities. Higher self-care behaviors were significantly associated with higher baseline confidence and lower distress. Those in the most disadvantaged positions (less education, less financial stability, and no health insurance) showed significantly larger improvement in self-care behaviors.

**Conclusions:**

Given the benefits associated with this study’s interventions, we suggest future work to further develop digital content that can be tailored to individuals with T2DM to help them manage their chronic conditions in a cost-effective manner.

## Introduction

### Background

Type 2 diabetes mellitus (T2DM) is complex, costly, and often preventable; yet, millions still live with this destructive disease, with many more Americans diagnosed annually. Ideally, incidence rates would be on a downward trend; however, this is not the case, with adult incidence rates remaining relatively stable over the past ~20 years, albeit with some variation following 2008 [1]. Thus, with more individuals diagnosed, coupled with this being a lifelong disease upon diagnosis, prevalence rates are on the rise, with an increase from 10% (2001‐2004) to 13% (2017‐2020) [1]. Therefore, this remains a critical national public health issue with additional evidence of differential patterns impacting individuals across both individual-level and place-based characteristics [2].

The United States is home to many residents living outside major metropolitan areas, who have historically faced inequities in accessing critical health care resources [3,4]. Recent estimates suggest that age-adjusted prevalence of diagnosed diabetes was higher among those residing in nonmetropolitan areas, with rates of 8.9% and 7.9% for both men and women, respectively, among large central metro areas, compared to rates of 10.5% and 8.6% in nonmetropolitan areas [1]. Furthermore, in terms of individual-level characteristics, inequities exist across characteristics such as age (with higher rates with increasing age) and sex (with higher rates among males) [1]. Thus, this is a complex and interrelated issue spanning multiple levels. Making matters worse, individuals with diabetes often face potentially preventable medical encounters due to, in part, poor management of diabetes, including higher risks of heart disease, stroke, blindness, kidney disease, amputations, and other items [5]. For many, these complications could potentially be prevented with proper maintenance of diabetes and related comorbidities, as applicable.

Effective self-management involves taking the necessary steps to monitor and maintain optimal glycemic levels and prevent adverse effects [6-8]. Additional components of effective self-management include medication adherence and lifestyle modifications (eg, diet or nutrition modifications, physical activity, and refraining from, or quitting, smoking) [9-11]. Thus, interventions aimed at reducing potentially preventable complications and improving the quality of life of individuals diagnosed with diabetes should consider both individual-level characteristics in combination with place-based measures, consistent with a strong theoretical foundation [12,13].

In terms of interventions, they can take several forms and platforms, including but not limited to online access to critical information; yet, there are some core elements to consider. For example, experts recommend all patients diagnosed with diabetes receive diabetes self-management education and support (DSMES) to provide them with the knowledge, resources, and skills essential to support their self-care journey and manage their condition [8]. Research indicates patients who receive DSMES are more likely to practice recommended self-care behaviors and clinical preventive care practices, such as routine clinical examinations and vaccines [9]. Additionally, positive changes in knowledge [14,15], confidence [8,16], and reduced distress [8,17] have been consistently reported and associated with sociodemographic and health factors, such as age, sex, education, race or ethnicity, rurality, financial stability, insurance coverage, and general health status [18-20].

With the rise in technological development and aftereffects of the COVID-19 pandemic, there is now a greater variety and availability of DSMES in the virtual and digital space. Interventions come in various formats from in-person to virtual (including both synchronous and asynchronous) to hybrid and contain various components or mechanisms of support, including, but not limited to, behavioral tracking and monitoring, peer support, coaching, knowledge-based resources, and skill building [21-23]. As no program is a one-size-fits-all, there are pros and cons to consider for both in-person and virtual DSMES programs.

Consistent with the diffusion of innovations theory [24], many people may be skeptical about the effectiveness of new digital health interventions (which include telehealth, telemedicine, mobile health, and eHealth [25]) and wary to engage in such practices. However, a systematic literature review and meta-analysis revealed that digital health interventions for people with T2DM were associated with significant improvements in hemoglobin A_1c_ levels [26]. Additionally, digital interventions may fill some gaps of in-person interventions, including accessibility, real-time feedback, and transportation barriers [27,28]. However, it is also important to note that users’ health literacy and/or digital literacy may impact their usage, level of engagement, and outcomes of using digital health interventions [29,30].

As new programs are developed, researchers have analyzed the acceptability, usability, and impacts of digital DSMES [7]. However, most studies analyzing the impacts and outcomes of DSMES compare the use of a digital program to a control group [7,31-35]. Additionally, studies tend to recruit from one community, limiting the generalizability of findings and the inability to observe the effectiveness of different digital health DSMES programs on behavioral outcomes across different geographic regions.

### Study Aims

This study, part of the Texas A&M University Rural Health Moonshot Initiative [6], attempted to address these research gaps by comparing the impact of 2 digital DSMES programs delivered separately and in combination to adults with T2DM across many settings in Texas. The objectives were to examine (1) the sociodemographic and general health characteristics of adults living with diabetes who agreed to participate in a randomized clinical trial and whether any of these baseline characteristics were significantly associated with group assignment which served as a test of our randomization protocol; (2) mean changes and effect size differences in knowledge, confidence, distress, and self-care activities by intervention and rurality status; and (3) whether knowledge, confidence, and distress, controlling for sociodemographic factors and general health status predicted changes in self-care activities over time.

## Methods

This study is reported in accordance with the CONSORT (Consolidated Standards of Reporting Trials) reporting guidelines for randomized trials (Checklist 1).

### Setting and Population

As described in detail in an earlier study [6], we recruited 188 adults aged 25 years and older living in Texas from November 2020 to March 2022 who reported having T2DM with baseline hemoglobin A_1c_ (HbA_1c_) levels of 7.5 or higher, indicating poorly managed diabetes and potential benefit from an intervention [36,37] targeted toward improving one’s management of diabetes. Recruitment efforts were geared toward participants from both rural and urban communities in Texas. Interested participants were considered eligible if they had access to a smartphone with internet connectivity and were able to read and speak English.

### Patient and Public Partnership or Involvement

This trial was generated based on the research team’s experiences of implementing diabetes management programming in rural and underserved communities [6,38,39]. Feedback was obtained from program implementers and participants in relation to program content, access, and attendance. Using this information obtained in the years preceding the trial, we updated the intervention content and delivery format accordingly. While originally attempting to test the in-person version of the intervention, the COVID-19 pandemic required us to pivot to virtual delivery. Representatives from community-based organizations, health care organizations, program facilitators, and past participants were engaged in the material development and testing phase of the digital translation. Furthermore, these representatives were engaged when attempting to identify the best recruitment and retention strategies, given high levels of attrition in past initiatives. Community and clinical partners were instrumental in participant recruitment efforts. While participants were not directly involved in the selection of measures, previously used subjective measures were included based on their success in related interventions and trials [6,38]. Findings were shared with the funder and community partners in accordance with their preferences. These took the form of informal meetings and discussions with stakeholders, flyers, virtual correspondence (eg, websites and social media), conference presentations, and publications.

### Recruitment Strategies

Knowing that multiple strategies would be needed to reach community-dwelling participants throughout the state, a variety of recruitment strategies were used, including radio advertisements, radio segments, flyers, bulletin boards, social media campaigns, directed letter campaigns from in-network health care providers, newspapers, email listserves, in-person recruitment, and the study’s website. We also used multiple retention strategies: participant incentives, distribution of study information, and frequent communication via text messaging, emails, and phone calls to limit attrition.

### Ethical Considerations

This study was reviewed and approved by the Texas A&M University Institutional Review Board (IRB 2019-0804D) and registered at ClinicalTrials.gov (ID NCT06370494). All participants provided written consent to participate in this randomized controlled trial (RCT). Participation was voluntary, and participants could withdraw from the study at any time without penalty. Deidentified data on major study outcomes were placed in the Texas A&M data repository upon completion of data analyses and report writing. Only the study team could access study data. Depending on their participation in the study, each participant could receive upward of US $375 for completing data throughout the study period.

### Interventions

Participants were randomized into 1 of 3 intervention arms, as described in the following paragraph. Each intervention aligned with American Diabetes Association national standards for diabetes self-management and best practices for behavioral change [40]. DSMES is a continuous process designed to help individuals with diabetes gain the knowledge, skills, and confidence needed to manage their condition effectively [41]. Each DSMES may vary in components, objectives, and mechanisms, but there is consensus that goals of DSMES are to promote informed decision-making, encourage healthy self-care habits, enhance problem-solving abilities, foster collaboration with health care providers, and ultimately improve health outcomes, overall well-being, and quality of life [41,42]. Furthermore, the American Diabetes Association and International Diabetes Federation established guidelines for DSMES programs to not only be reimbursable but also recognized and accredited as evidence-based and effective programs.

Differing in modality, the 3 intervention arms (further described in Table 1) included (1) Virtual Making Moves with Diabetes (vMMWD) Group, (2) Technology-Based Education and Support (TBES) Group, and (3) a combined approach of vMMWD and TBES. Participants from all groups received an HbA_1c_ monitoring kit, a glucose meter, and supplies to test their blood sugar at home. On the basis of the prior literature documenting positive effects of DSMES [8,9,16,17,21-23,43-46], we hypothesized that all intervention strategies would have a beneficial effect on diabetes-related study outcomes, but that effects would be stronger for vMMWD over TBES, given the greater personalization, and that the combined intervention strategy (representing the strengths of each intervention modality) would have the strongest effects. We also hypothesized that participants from traditionally underserved and resource-limited settings (eg, minority identity, less education, lower financial security, no health insurance, and rural residence) who typically have less access to diabetes self-care programs might have the greatest improvements.

**Table 1.** Overview of interventions.

Intervention group	Description
vMMWD^a^ group	Asynchronous virtual education and support program with one-on-one follow-up counseling with a diabetes specialist. Entailed 3 education sessions within 3 mo (totaling about 6‐8 h). Also entailed one-on-one interactions with a registered nurse or registered dietitian to personalize content, build upon virtual education sessions, and create tailored diabetes self-care strategies.
TBES^b^ group	Smartphone app to provide continuous, but less structured, support. Participants used the app at their own pace to learn and practice diabetes self-care skills and strategies. The TBES included a chat feature for participants to get personalized support from a diabetes coach.
Combination group	Combined modality where participants sequentially received vMMWD and TBES (upon completion of vMMWD) to maximize support and behavioral reinforcement.

a vMMWD: Virtual Making Moves with Diabetes.

b TBES: Technology-Based Education and Support.

### Measures

We selected standardized measures that have been previously used in diabetes education studies, as described below.

1. Diabetes knowledge: We used a variant of the Spoken Knowledge in Low Literacy in Diabetes Scale used in prior diabetes self-management studies [47,48]. Our knowledge measure was composed of 10 true or false knowledge questions, such as “Glycosylated hemoglobin (HbA_1c_) is a test that measures your average blood sugar level in the past week” and “Having regular check-ups with your healthcare provider can help spot the early signs of diabetes complications.” The total score represented the percentage of correctly answered questions.
2. Diabetes confidence: This was measured using the 8-item Self-Efficacy for Diabetes scale, rated on a 10-point scale from 1 (“not at all confident”) to 10 (“totally confident”), with values ranging from 8 to 80 points, with higher points demonstrating better confidence in diabetes self-care management [49]. The diabetes self-efficacy scale measured participants’ confidence to engage in self-care behaviors, such as eating meals every 4 to 5 hours, exercising for 15 to 30 minutes, 4 to 5 times a week, following their diet when they have to prepare or share food with other people who do not have diabetes, and knowing what to do when their blood glucose level goes higher or lower than it should be. An example item was as follows: “How confident do you feel that you can control your diabetes so that it does not interfere with the things you want to do?”
3. Diabetes distress: The Diabetes Distress Scale [50] was used to measure distress in 4 domains: emotional burden, physician-related distress, regimen-related distress, and interpersonal distress (eg, “Feeling that I am not testing my blood sugars frequently enough”). Six diabetes-specific items were rated on a 6-point Likert scale, ranging from 1 (“not a problem”) to 6 (“serious problem”), with the scale ranging from 6 to 36 points. Reverse coding was not required, and a higher mean score of 6 items indicates greater levels of diabetes-related distress.
4. Diabetes self-care behaviors: We used 9 items from the Summary of Diabetes Self-Care Activities measure [51] based on the mean number of days engaged in different behaviors, such as general diet, specific diet, exercise, blood glucose testing, and foot care. The scale ranged from 0 to 63, with higher scores reflecting better engagement in key self-care behaviors. This scale has been shown to be a brief yet reliable and valid self-report measure of diabetes self-care used in both research and practice.
5. Sociodemographic and health-related variables: We included standard self-reported demographic measures of age, sex, race (White vs non-White), ethnicity (Hispanic or Latino vs not), education (more than high school vs high school or less), insurance coverage (yes vs no), and financial stability (yes vs no).
6. Place-based variables: Additionally, we characterized participants’ settings as rural versus urban. The National Center for Health Statistics Urban–Rural Classification Scheme for Counties [52] was used to dichotomize participants into metropolitan (levels 1‐4) and nonmetropolitan areas (levels 5‐6) consistent with our aims of examining outcomes from both urban and rural areas.
7. Self-rated health: We also included a measure of self-rated health, asking participants to rate their health as excellent, very good, good, fair, or poor and grouped responses into 3 groups: excellent, very good or good, and fair or poor [53].

### Study Design and Analyses

#### Overview

The team implemented an RCT to evaluate the relative effectiveness of three approaches: (1) vMMWD alone, (2) TBES alone, and (3) a combined sequence in which participants first received vMMWD followed by TBES. Each intervention was delivered over a 3-month period, with data collected at baseline and at 3- and 6-month follow-up. A 2-stage randomization process was used to allocate participants to the respective intervention arms, and no dropout was observed between the first and second randomization stages. Prior to study initiation, sample size calculations were performed for the primary outcome measure, HbA_1c_. Expected changes in HbA_1c_ were informed by findings from an earlier South Texas study using a similar intervention [38,39]. On the basis of an assumed moderate effect size (Cohen *d*=0.5), power analyses indicated that a minimum of 34 participants completing both baseline and postintervention assessments would be required to detect statistically significant differences in HbA_1c_, with 80% power and a 2-sided paired *t* test at the 0.05 significance level. Assuming that effect sizes would be even larger for the self-care intervention impacts, the sample size was deemed adequate to detect statistically significant differences in self-care behaviors. As no nonintervention control group was included, all effectiveness analyses are interpreted as within-group changes over time. For between-group comparisons, the TBES-only group was treated as the reference category in regression models due to its lower intensity relative to other modalities.

#### Randomization Procedures

As described previously [6], the randomization involved 2 stages. The first stage was an individual-based block-stratified randomization into 1 of 2 arms, vMMWD only. or TBES only, with a ratio of 2:1, with 2 participants in vMMWD relative to 1 participant in TBES, which was stratified by rural and urban areas with a block size of 3. The second stage of randomization was conducted for the participants who completed vMMWD in the first stage. It was an individual-based block randomization for 1 of 2 arms: vMMWD only (ie, no additional intervention) versus combined modality (ie, receive TBES after they already received vMMWD), with a ratio of 1:1. Furthermore, in designing the study, we debated whether the combined treatment arm should be concurrent or sequential. For both logistical and conceptual reasons, we decided to phase in the TBES condition after the more personal vMMWD intervention, with the assumption that the app condition would help sustain any benefits achieved in a time-limited intervention.

All 3 intervention modalities were conducted for 3 months. Knowledge, confidence, distress, and self-care activity scores were observed at baseline and follow-up periods at 3 and 6 months.

#### Descriptive Analyses

As described in Ory et al [6] for the analysis of HbA_1c_, an intent-to-treat analysis was also conducted for all the behavioral variables. Descriptive statistics were calculated for all participants by intervention arm. We provided medians with IQRs for continuous variables, and frequencies with percentages for discrete variables. Wilcoxon rank-sum tests and Pearson chi-square tests were used to test associations between low versus high baseline self-care and the covariates in Table 2 and regression.

Changes in knowledge, confidence, distress, and self-care activities were calculated as differences between baseline and 3-month follow-up and between baseline and 6-month follow-up (eg, “3 mo minus baseline” and “6 mo minus baseline”). For knowledge, confidence, distress, and self-care activity, the average changes and 95% CIs of mean changes based on *t* distributions were calculated for all participants, for participants in each study arm, and for participants residing in rural and urban areas. Using the average and SD of changes, we calculated and reported Cohen *d* from the paired *t* test as effect size.

#### Longitudinal Analyses

Longitudinal self-care activity scores at baseline and at 3-month and 6-month follow-ups were modeled as a longitudinal outcome using knowledge, confidence, and distress measured at baseline and follow-ups, as well as covariates from Table 2 measured only at baseline. The follow-up time (operationally defined as 0 at baseline, 3 at 3-month follow-up, and 6 at 6-month follow-up) was also a covariate in the analysis, which indicated whether the change of the outcome score was significant after adjusting for all the covariates. The fully adjusted model included follow-up time, knowledge, confidence, distress, treatment modality, and study demographic and health variables. The coefficients and *P* values of knowledge, confidence, and distress indicate the direction and significance of associations between these variables and the outcome variable. The longitudinal analysis with generalized estimating equations was implemented using SAS software (SAS Institute), the GENMOD procedure. Results from Tables 2–4 were generated using SAS software, version 9.4. A *P* value of .05 or less was considered statistically significant.

#### Causality

Random assignment was used to increase internal validity in terms of the intervention effects [54]. This increased our likelihood of identifying a true effect, considering the study design, albeit not without some limiting factors (see section *Limitations*).

## Results

**Figure 1.**
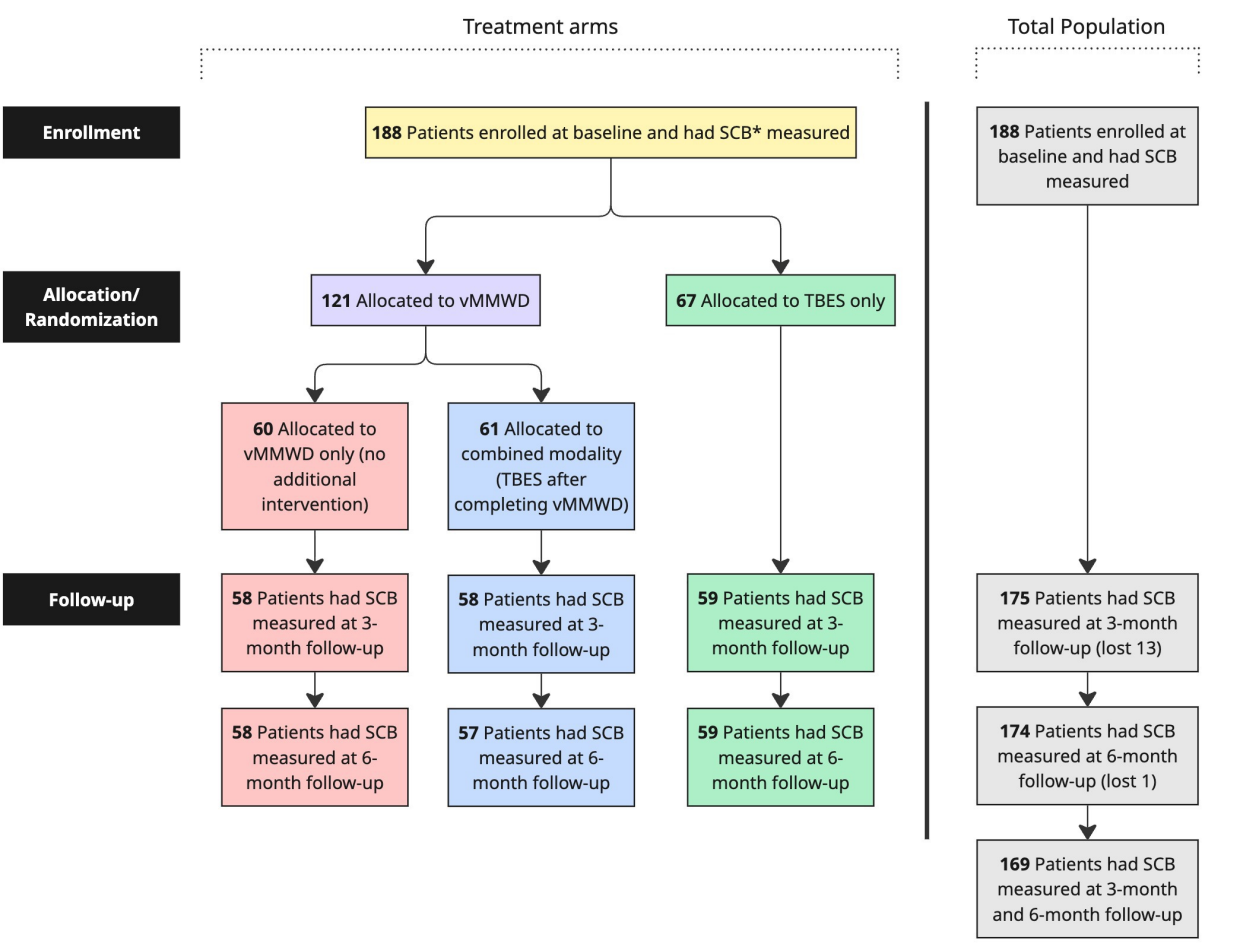
Participant flow diagram. SCB: self-care behavior; TBES: Technology-Based Education and Support; vMMWD: Virtual Making Moves with Diabetes.

There was minimal participant attrition over time. As indicated in the CONSORT diagram (Figure 1), of the 188 participants enrolled at baseline, there was no drop off between enrollment and randomization. Concerns about selection bias based on the 2-stage randomization process were minimized because there were no significant differences between vMMWD completers and noncompleters prior to stage 2 randomization. Furthermore, the amount of missing data on the key dependent variable at 3-month and/or 6-month follow-ups was also minimal (ie, no more than 10%), mitigating the need for data imputation. Table 2 presents sample characteristics by intervention arm. In terms of the intervention arm, 32% (60/188) were in the vMMWD only condition, 36% (67/188) were in the TBES only condition, and 32% (61/188) were in the combined vMMWD and TBES condition. The median age of participants was 52 years (IQR 44-59), with most participants self-identifying as female (146/189, 77%), non-Hispanic or Latino (116/189, 61%), and White (163/189, 86%), and having more than a high school education (149/188, 79%). More than half of the participants (104/189, 55%) reported being in poor or fair health at baseline. At baseline, most participants reported being financially stable (160/189, 85%) and having health insurance (147/171, 86%). When comparing sample characteristics by intervention arm, no significant associations emerged (*P*>.05).

**Table 2.** Sample characteristics by intervention arms.

Variables and level	All	vMMWD^a^	TBES^b^	Combined	*P* value
Age (y), median (range), n	52 (44-54), 189	50 (43-55), 60	53.5 (46.5‐59.5) 68	53 (43-54), 61	.27
Sex, n (%)					
Female	146 (77)	47 (78)	54 (79)	45 (74)	.73
Male	43 (23)	13 (22)	14 (21)	16 (26)	
Ethnicity, n (%)					
Hispanic/Latino	73 (39)	25 (42)	23 (34)	25 (41)	.59
Not Hispanic/Latino	116 (61)	35 (58)	45 (66)	36 (59)	
Binary race, n (%)					
Non-White	26 (14)	11 (18)	7 (10)	8 (13)	.41
White	163 (86)	49 (82)	61 (90)	53 (87)	
General health, n (%)					
1=poor/fair	104 (55)	33 (55)	36 (53)	35 (57)	.17
2=very good/good	64 (34)	22 (37)	27 (40)	15 (25)	
3=excellent	21 (11)	5 (8)	5 (7)	11 (18)	
Rural status, n (%)					
Rural	67 (35)	22 (37)	23 (34)	22 (36)	.94
Urban	122 (65)	38 (63)	45 (66)	39 (64)	
Financial stability, n (%)					
0=no	29 (15)	10 (17)	10 (15)	9 (15)	.94
1=yes	160 (85)	50 (83)	58 (85)	52 (85)	
Education, n (%)					
0=less than high school	39 (21)	9 (15)	14 (21)	16 (26)	.31
1=greater than high school	149 (79)	51 (85)	53 (79)	45 (74)	
Have insurance, n (%)					
1=yes	147 (86)	50 (86)	50 (86)	47 (85)	.99
2=no	24 (14)	8 (14)	8 (14)	8 (15)	
Knowledge baseline, median (range), n	8 (7-9), 188	8 (7-9), 60	9 (7‐9.5), 68	8 (6-9), 60	.23
Distress baseline, median (range), n	22 (14-28), 189	22 (14.5‐29), 60	21 (14-27), 68	23 (15-27), 61	.79
Confidence baseline, median (range), n	45 (35-56), 189	41.5 (32.5‐51.5), 60	49 (36‐57.5), 68	45 (35-53), 61	.10
Self-care behavior baseline, median (range), n	26 (17-35), 188	21 (16.5‐35.5), 60	27 (18-36), 67	27 (18-34), 61	.39

a vMMWD: Virtual Making Moves with Diabetes.

b TBES: Technology-Based Education and Support.

Table 3 reports mean changes in outcome variables overall and by rural status and intervention arm. Effect sizes are also reported, reinforcing that we were sufficiently powered to detect statistically significant differences. Overall, for all participants combined, significant improvements were observed from baseline to 3-month and 6-month follow-up for knowledge, confidence, distress, and self-care activities (*P*<.001). All but one subgroup (ie, the TBES only intervention arm) showed significant improvements from baseline to 3-month and 6-month follow-up for knowledge (*P*<.001). Among all geographic statuses and intervention arms, significant improvements were observed from baseline to 3-month and 6-month follow-up for confidence, distress, and self-care activities (*P*<.05). Effect sizes for the total population ranged from 0.446 for knowledge to 0.827 for self-care activities at the 3-month follow-up and from 0.538 for knowledge to 0.888 for self-care activities at the 6-month follow-up assessment.

**Table 3.** Mean differences in outcome variables for all participants and by rural status and intervention arm.

Outcome variables	3 mo−baseline		6 mo−baseline	
	Mean Δ (95% CI) [n]	Effect size^a^	Mean Δ (95% CI) [n]	Effect size
Knowledge				
Total	0.769 (0.512 to 1.026)^b^ [173]	0.446	0.866 (0.625 to 1.107)^b^ [172]	0.538
Rural	0.921 (0.531 to 1.311)^b^ [57]	0.583	0.922 (0.493 to 1.350)^b^ [58]	0.527
Urban	0.682 (0.345 to 1.019)^b^ [110]	0.378	0.833 (0.544 to 1.122)^b^ [108]	0.544
TBES^c^	0.085 (−0.387 to 0.557) [54]	0.046	0.373 (−0.003 to 0.749) [54]	0.253
vMMWD^d^	1.086 (0.731 to 1.441)^b^ [55]	0.787	1.121 (0.720 to 1.521)^b^ [55]	0.720
Combined	1.161 (0.712 to 1.610)^b^ [59]	0.677	1.127 (0.675 to 1.579)^b^ [60]	0.659
Confidence				
Total	8.520 (6.211 to 10.830)^b^ [171]	0.553	12.127 (9.724 to 14.530)^b^ [173]	0.752
Rural	5.968 (2.051 to 9.884)^e^ [61]	0.379	11.125 (6.907 to 15.343)^b^ [58]	0.646
Urban	9.972 (7.138 to 12.807)^b^ [109]	0.661	12.716 (9.806 to 15.626)^b^ [109]	0.820
TBES	4.138 (0.029 to 8.247)^e^ [55]	0.259	7.864 (4.203 to 11.526)^b^ [54]	0.548
vMMWD	13.228 (9.418 to 17.038)^b^ [62]	0.901	15.190 (11.418 to 18.961)^b^ [55]	1.037
Combined	8.268 (4.501 to 12.035)^b^ [59]	0.575	13.446 (8.600 to 18.293)^b^ [59]	0.727
Distress				
Total	−4.392 (−5.477 to −3.307)^b^ [171]	0.607	−5.509 (−6.913 to −4.106)^b^ [106]	0.748
Rural	−4.145 (−5.863 to −2.428)^b^ [61]	0.601	−4.500 (−7.011 to −1.989)^b^ [30]	0.641
Urban	−4.532 (−5.931 to −3.133)^b^ [109]	0.608	−5.908 (−7.597 to −4.219)^b^ [63]	0.786
TBES	−2.517 (−4.321 to −0.714)^e^ [55]	0.359	−3.568 (−6.020 to −1.115)^e^ [37]	0.469
vMMWD	−6.404 (−8.363 to −4.444)^b^ [62]	0.848	−7.353 (−9.767 to −4.939)^b^ [34]	1.024
Combined	−4.286 (−6.046 to −2.526)^b^ [59]	0.638	−5.771 (−8.086 to −3.456)^b^ [35]	0.826
Self-care activities				
Total	9.994 (8.199 to 11.790)^b^ [174]	0.827	10.295 (8.568 to 12.022)^b^ [173]	0.888
Rural	9.143 (5.822 to 12.464)^b^ [57]	0.680	11.125 (8.350 to 13.900)^b^ [58]	0.982
Urban	10.477 (8.381 to 12.574)^b^ [111]	0.930	9.807 (7.599 to 12.016)^b^ [109]	0.834
TBES	8.431 (5.502 to 11.360)^b^ [55]	0.741	7.638 (4.561 to 10.715)^b^ [55]	0.639
vMMWD	12.759 (9.801 to 15.717)^b^ [55]	1.110	12.948 (9.765 to 16.132)^b^ [55]	1.047
Combined	8.793 (5.440 to 12.146)^b^ [55]	0.675	10.298 (7.746 to 12.850)^b^ [62]	1.048

a The effect size is the one-sample Cohen *d*.

b Statistical significance from testing if the change was different from 0 if the *P* value is less than .001.

c TBES: Technology-Based Education and Support.

d vMMWD: Virtual Making Moves with Diabetes.

e Statistical significance from testing if the change was different from 0 if the *P* value is between .001 and .05.

Table 4 reports findings from the longitudinal analysis examining factors associated with higher self-care activity scale scores at follow-up. The model was adjusted for the primary independent variable, follow-up time, and covariates, such as knowledge, confidence, distress, sociodemographic variables, and self-assessed health. On average, participants reported significantly higher self-care activity scores from baseline to follow-up (estimate=0.93; *P*<.001). Participants who reported higher baseline confidence scores (estimate=0.30; *P*<.001) and lower baseline distress scores (estimate=−0.50; *P*<.001) reported significantly higher self-care activity scores at follow-up, respectively. Participants who reported having less education (estimate=−3.10; *P*=.04), no health insurance (estimate=−3.35; *P*=.047), and lower financial stability at baseline (estimate=−3.93; *P*=.01) reported significantly higher self-care activity scores at follow-up, respectively. However, self-care activities were not significantly associated with diabetes knowledge or intervention modality (all *P*>.05).

**Table 4.** Longitudinal regression analysis of self-care activities with knowledge, confidence, distress, and covariates.

Parameters^a^	Estimate^b^	SE	95% CI		*P* value
Upper limit	Lower limit
Follow-up time				
From baseline to 6 mo	0.93	0.20	0.54	1.32	<.001
Knowledge					
Higher score reflects more correct answers	−0.37	0.39	−1.13	0.40	.35
Confidence					
Higher score reflects more confidence	0.30	0.05	0.20	0.39	<.001
Distress					
Higher score reflects more distress	−0.50	0.09	−0.68	−0.32	<.001
Age					
Continuously measured	0.02	0.07	−0.11	0.15	.76
Sex					
Female (vs male)	0.00	1.63	−3.18	3.19	.998
Ethnicity					
Hispanic (vs non-Hispanic)	0.05	1.49	−2.86	2.97	.97
Race					
White (vs non-White)	−0.74	1.92	−4.50	3.02	.699
Rural status					
Urban (vs rural)	−0.81	1.31	−3.37	1.75	.54
Education					
More than high school (vs high school or less)	−3.10	1.54	−6.11	−0.08	.04
General health					
Very good or good (vs fair or poor)	1.47	1.51	−1.49	4.43	.33
Excellent (vs fair or poor)	1.89	2.14	−2.29	6.08	.38
Arm					
Combined (vs app)	0.14	1.61	−3.01	3.29	.93
Class (vs app)	−0.83	1.44	−3.65	2.00	.57
Financial stability					
Ends meet (vs not met)	−3.93	1.61	−7.08	−0.78	.01
Insurance					
Yes (vs no)	−3.35	1.69	−6.66	−0.05	.047

a Referent levels are listed in parentheses.

b The positive estimate of time and confidence and negative estimate of distress are associated with improved self-care activities.

## Discussion

### Principal Findings

This study examined the comparative effectiveness of 3 digital DSMES interventions among adults with T2DM, with a particular focus on differences by intervention modality and sociodemographic factors. Findings demonstrated that (1) randomization was effective in creating 3 distinct intervention groups (objective 1); (2) all intervention modalities significantly improved diabetes-related confidence, distress, and self-care behaviors over time, with sustained effects (objective 2); and (3) confidence and distress, controlling for sociodemographic factors and general health status, predicted significant changes in self-care activities over time (objective 3). Importantly, no statistically significant differences were observed between intervention modalities, and improvements were largely consistent across rural and urban participants, a major covariate.

To our knowledge, this study is among the first to rigorously evaluate and directly compare different digital diabetes self-management programs using an RCT design in this way. All 3 interventions—vMMWD, TBES, and the combined approach—led to significant improvements in diabetes-related confidence, distress, and self-care behaviors, with effects through 6 months and supported by moderate to strong effect sizes. These findings generally reflect positive findings reported in other in-person and digital health diabetes interventions [8,9,16,17,21-23,43-46]. However, the TBES intervention did not result in significant improvements in diabetes-related knowledge from baseline to follow-up, contrary to other research showing that coaching-based apps can significantly increase self-management diabetes knowledge.

While all interventions demonstrated overall positive outcomes, no statistically significant superiority was observed among the intervention modalities, consistent with HbA_1c_ outcome findings reported elsewhere [6,55]. These results offer partial support for the study’s hypotheses, confirming the effectiveness of each intervention but not the expected added benefit of the combined approach. This suggests the observed improvements were likely due to shared attributes (ie, DSMES components) rather than the intervention modality. Thus, whichever modality is easily accessible to participants is likely to benefit their self-care behaviors.

The study findings also partially supported our expectations that those from underserved or low-resourced settings with greater need would experience stronger positive effects, providing evidence for the heterogeneous treatment effect [56]. However, with regard to rurality, participants in both rural and urban settings experienced significant improvements across outcomes, with no statistically significant differences observed between groups. While rural participants showed comparable—if not slightly larger—gains in some outcomes (eg, knowledge and self-care), the study was not sufficiently powered to detect interaction effects. These findings suggest that digital DSMES may help mitigate geographic disparities in access to diabetes education.

The study findings can be interpreted through the lens of access, cost, and quality in diabetes self-management. From a quality perspective, all 3 interventions demonstrated encouraging effectiveness. Our prior research [6] demonstrated improvements in HbA_1c_ values, whereas this follow-up research revealed positive impacts on psychosocial factors linked to clinical outcomes [57,58]. Specifically, our DSMES-based interventions significantly improved psychological outcomes—such as confidence and distress—as well as actual self-care behaviors. These results suggest that the interventions enhanced participants’ understanding and confidence in managing their diabetes, reduced diabetes-related distress, and facilitated real behavioral changes. As emphasized in classic models of chronic disease management, sustained behavioral improvements often arise from underlying psychological shifts—particularly in self-efficacy and emotional readiness [59]—which are foundational in effective chronic illness care [60].

These concepts are further supported by the Chronic Care Model [61], which underscores the importance of informed, activated patients and prepared, proactive health care teams. Prior research supports the notion that self-management programs are most effective when they improve knowledge and confidence while alleviating distress [8,41,62]. Importantly, this study also offers evidence of sustainability, with program effects maintained from baseline through 3 and 6 months. This is especially encouraging, given previously mixed findings in the literature regarding the long-term effectiveness of digital diabetes self-management programs [63,64].

The longitudinal regression findings can also be interpreted as supporting the social cognitive theory. The positive association between confidence and self-care behaviors is consistent with evidence that self-efficacy is a key driver of sustained behavior change [59,60], while the negative effect of diabetes-related distress reflects well-documented emotional barriers that undermine adherence and self-management capacity [50,62]. Together, these results reinforce that behavior change is not driven by knowledge alone, but by psychosocial mechanisms that enable individuals to translate knowledge into action. By demonstrating that confidence and distress—but not knowledge—predict longitudinal improvements in self-care, this study strengthens the theoretical understanding of how DSMES interventions influence behavior over time.

From an access perspective, this study highlights the feasibility and value of digital diabetes self-management programs. Accelerated by advancements in digital technology and the societal shifts prompted by the COVID-19 pandemic, there is increasing acceptance of accessing health care services via digital platforms [65]. Given persistent barriers to in-person care—particularly in rural areas where geographic isolation, limited transportation options, and inadequate health care infrastructure prevail—these findings support the use of digital programs as a viable alternative to traditional in-person services [66,67].

A prior meta-analysis further supports the potential of digital diabetes self-management education to improve clinical and behavioral outcomes while also highlighting significant variability in program implementation, user engagement, and access across populations [63]. Policymakers, public health professionals, and individuals with diabetes should consider DSMES as a practical and scalable solution, provided that participants have access to the necessary devices and internet connectivity. Notably, leveraging community resources—such as public libraries with Wi-Fi and private telehealth spaces—could bridge the digital divide and extend digital health access in underserved communities [68]. From a cost perspective, self-management remains a critical pillar of diabetes care, with substantial evidence supporting both the effectiveness and cost-efficiency of DSMES [8,69]. Although this study did not directly assess the cost of digital DSMES, its findings contribute to the growing literature supporting the economic and practical value of digital delivery methods. Digital interventions can reduce or eliminate common logistical barriers—such as travel time, facility utilization, and provider burden—making them particularly advantageous for underserved and rural populations. Prior studies have shown that telehealth-based diabetes interventions significantly enhance access to care and treatment adherence in rural settings by minimizing travel burden and expanding provider reach [70-72].

Future research steps include examining the mechanisms through which digital interventions improve diabetes outcomes—particularly the interrelationships between knowledge, confidence, emotional distress, and self-care behaviors. A deeper understanding of these pathways can inform the design of more targeted, effective interventions. Ultimately, digital DSMES programs hold promise not only for improving health outcomes (quality) but also for increasing accessibility to care and reducing the economic burden associated with diabetes management—advancing the triple aim of better care, improved population health, and lower costs [73].

### Limitations

This study has several limitations typical in single-site randomized clinical trial studies. First, the sample was limited to one state (Texas), which may affect the generalizability of findings to other geographic regions. However, this state includes a large and geographically diverse population (including an international border, many rural counties, and several major metropolitan areas). Furthermore, Texas is located within the South census region with a history of high rates of diabetes [2,4], with other work even pointing to higher diabetes mortality rates relative to other census regions [74]. Thus, Texas is a unique area, yet also representative of various sociodemographic groups.

Second, we were not able to recruit as many participants from rural areas as we had projected, constraining our ability to conduct formal subgroup analyses. While preimplementation sample size estimates indicated adequate power for testing main effects of DSMES programs with moderate or larger effect sizes, the relatively small sample size within each intervention arm may have limited the ability to detect differences between rural and urban subgroups. Hence, we were not able to distinguish relative intervention benefits for rural versus urban populations.

Third, all participants were required to have internet access and a smartphone, potentially introducing selection bias and limiting generalizability to those with lower digital literacy or access. However, according to a Pew Research Survey, the percentage of Americans who “rely on their smartphones for going online” (percentage of adults with a smartphone, but no broadband at home) has seen increases across all income categories from 2013 to 2021 [75]. In fact, the largest gains were for those with household incomes of less than US $30,000 (the lowest income level in the study) from 12% to 27% from 2013 to 2021 [75]. Thus, this is actually a strength, in that this method (eg, use of a smartphone to access diabetes-related training) is likely to gain more momentum over time with more Americans becoming more familiar with their smartphones as a major means of accessing information, etc. In fact, more than 90% of study participants reported being comfortable with smartphones, minimizing concerns about digital literacy in this group of participants. Yet, as indicated earlier, our study participants may not be generalizable to the broader population of people living with diabetes who may have lower digital literacy and underscore equity concerns needing policy attention [76,77].

Fourth, all outcome measures were based on self-report, which may be subject to recall or social desirability bias. However, this is not uncommon in survey research and is applicable to even large nationally representative surveys such as the BRFSS [78].

Fifth, the study did not collect data on participants’ actual engagement with the digital interventions, limiting insights into usage patterns and dose-response relationships. However, the introduction of random assignment was a major strength that likely led to greater internal validity [54].

Finally, although our study had a 6-month follow-up period revealing short-term maintenance effects, a longer follow-up period, while not feasible during our study period, would have addressed the extent to which benefits from different digital interventions could be maintained over time.

### Conclusions

This RCT demonstrates the effectiveness of 3 digital DSMES interventions—vMMWD, TBES, and a combined modality—in improving diabetes-related confidence, reducing distress, and enhancing self-care behaviors among adults with T2DM. While most intervention groups showed improvements through 6 months on all diabetes-related outcomes, no single modality proved statistically superior. One notable exception emerged; knowledge gains were not significant in the TBES-only group. These findings underscore the value of digital DSMES as a flexible and scalable approach to support behavior change. Importantly, the study highlights how digital interventions can promote health equity by expanding access to self-care education for individuals in underserved and rural settings. Although the study did not directly assess costs, the digital format’s ability to reduce logistical barriers, such as travel, provider burden, and infrastructure demands, suggests strong potential for cost-efficiency.

These findings have important implications for public health practice and health care delivery. Digital DSMES programs represent a scalable and adaptable approach to supporting individuals with diabetes, particularly in underserved and resource-limited settings. Future research should explore the mechanisms linking knowledge, confidence, and distress to behavioral outcomes to optimize digital delivery strategies. Furthermore, given recent advancements in artificial intelligence, we also expect additional improvements in the user interface (including how individuals access digital data) on smartphones with potential opportunities for subject-specific (where applicable) tailoring of knowledge and other outcomes to improve the user experience in the future. For example, digital DSMES programs and platforms could incorporate adaptive messaging that responds to users’ ongoing data, such as increasingly motivational prompts and action steps when confidence scores decline [79]. Future research should focus on identifying the specific components and mechanisms that drive intervention effectiveness, including the roles of engagement, personalization, and behavioral support. Taken together, this work supports the promise of digital DSMES in advancing the goals of improved care quality, enhanced access, and reduced costs—aligned with the broader aim of transforming chronic disease management.

## Supplementary material

10.2196/87364 Checklist 1 CONSORT checklist.

## Abbreviations:

CONSORT: Consolidated Standards of Reporting Trials
DSMES: Diabetes Self-Management Education and Support
HbA_1c_: hemoglobin A_1c_
RCT: randomized controlled trial
T2DM: type 2 diabetes mellitus
TBES: Technology-Based Education and Support
vMMWD: Virtual Making Moves with Diabetes
